# Everolimus-induced pulmonary toxicity: Findings on ^18^F-FDG PET/CT imaging

**DOI:** 10.1097/MD.0000000000012518

**Published:** 2018-10-05

**Authors:** Sebastien Dejust, David Morland, Claire Bruna-Muraille, Jean-Christophe Eymard, Gabriel Yazbek, Aude-Marie Savoye, Dimitri Papathanassiou

**Affiliations:** a Department of Nuclear Medicine, Jean Godinot Institut; b Laboratory of Biophysics, University of Reims; c Department of Oncology, Jean Godinot Institut; d Research Center in Information and Communication Sciences and Technologies, EA 3804, University of Reims, Reims, France.

**Keywords:** everolimus, fluoro-deoxy-glucose, lung, positron emission tomography/computerized tomography, toxicity

## Abstract

The everolimus–exemestane combination is indicated in advanced breast cancer treatment and usually well tolerated. The objective of the study was to determine the frequency of everolimus lung side effects and investigate their imaging characteristics on positron emission tomography with 18F-fluoro-deoxy-glucose combined with computerized tomography (^18^F-FDG PET/CT).

Our single-center retrospective descriptive study systematically included all patients with metastatic breast cancer treated by this combination (n = 29 representing 57 ^18^F-FDG PET/CT). Number of segments involved was quantified. Maximum standardized uptake value (SUVmax), average standardized uptake value (SUVmean), metabolic target volume (MTV), and total lesion glycolysis (TLG) were measured. Severe pneumopathy was studied by subgroup analysis.

Pleuroparenchymal anomalies rate detected on ^18^F-FDG PET/CT was 62%. Alveolar-interstitial lesions were mainly observed (89%) and affected 2.8 segments (0.5–11.5) with a median of 2 segments. S7 and S10 were the most involved segments with SUVmax 3.9 (1.3–8.8) and SUVmean 2.2 (0.7–4.9). Statistically significant difference (*P* = .02) was found with number of segment involved to characterize severe pneumopathy (average of 6.3 segments [2.5–11.5] vs 1.9 segments [0.5–8] for interstitial lung disease) but not with SUVmax, SUVmean, MTV, TLG (*P* = .14, 0.22, 0.22, and 0.17, respectively).

The ^18^F-FDG PET/CT could highlight pulmonary everolimus side effects, with a typical imaging pattern: alveolar-interstitial opacities associated with moderate uptake, more or less extensive, mainly affecting the lower lobes. Rarely, a pseudotumoral aspect may be detected, corresponding to a pitfall. MTV or TLG showed a tendency to differentiate severe pneumopathy vs interstitial lung disease but no statistically significant differences was observed contrarily to the number of segments involved. Further studies are necessary to determine if the ^18^F-FDG PET/CT could early predict adverse effects of mTOR inhibitors.

## 1 Introduction

Novel targeted molecular therapies, among which Mamalian target of rapamycin (mTOR) inhibitors (including everolimus, sirolimus, temsirolimus, deforolimus) showed efficacy in oncology, especially in breast cancer when used alone or when combined with other therapies.^[1,2]^ The everolimus–exemestane combination is indicated in advanced breast cancer treatment with positive hormonal receptor, HER2/neu negative, in case of recurrence or progression of the disease in postmenopausal women without symptomatic visceral disease and previously treated with a nonsteroidal aromatase inhibitor, because of improvement of progression-free survival.^[3–5]^ One of the mechanisms involved in tumor resistance of first-line treatments (hormone therapy or immunotherapy) may be a permanent activation of intracellular pathway phosphatidylinositol 3-kinase (PI3K)/protein kinase B (AKT)/mTOR.^[6,7]^ By selectively blocking signal transduction, mTOR inhibitors can restore the sensitivity to hormonal therapy promoting the effectiveness of exemestane.^[8]^

Pulmonary side effects of selective inhibitors of mTOR (including everolimus)^[9,10]^ represent a class effect, that is common effects of all rapamycin derivatives.^[11]^ They were mainly studied by conventional imaging (radiography and computerized tomography [CT])^[12,13]^ but not yet evaluated by positron emission tomography with 18^F^-fluoro-deoxy-glucose combined with computerized tomography (^18^F-FDG PET/CT). ^18^F-FDG PET/CT acquired a central role in oncology^[14]^ particularly in breast cancer (initial staging, detection of recurrence, and evaluation of therapy response in case of metastatic disease). ^18^F-FDG PET/CT can also diagnose many inflammatory or infectious diseases.^[15]^ Nowadays, no study evaluated lung toxicity of mTOR inhibitors with ^18^F-FDG PET/CT. The objective of the study was to determine the frequency of everolimus lung side effects in breast cancer and investigate their imaging characteristics in ^18^F-FDG PET/CT.

## 2 Materials and methods

### 2.1 Patient eligibility

Our single-center retrospective descriptive study included patients with metastatic breast cancer initially treated by association of everolimus (10 mg/d) and exemestane (25 mg/d), similar to the dosage used in clinical practice, from 2012 to 2016, and referred for at least one ^18^F-FDG PET/CT in our center. All patients performed regular follow-up, consisting in a consultation for early toxicity detection 1 month after treatment initiation, then a quarterly clinical evaluation to assess the effectiveness and tolerance of the treatment, including a ^18^F-FDG PET/CT and blood biomarker dosage. The management of adverse events depended on the severity: grade I (asymptomatic, radiographic findings only): close monitoring; grade II (symptomatic but not interfering with activities of daily life): dosage adaptation (7.5 mg/d or 5 mg/d); grade III (symptomatic, interfering with activities of daily life, oxygen indicated); or grade IV (life-threatening, ventilatory support indicated): pause then half dose after resolution of the symptoms.

### 2.2 ^18^F-FDG PET/CT

Checking of fasting for at least 4 hours and capillary blood glucose before injection. Image acquisition approximately 60 minutes after radiotracer injection. The injected activity and image acquisition protocol varied with PET/CT camera used:

- Discovery 710 (General Electric, Milwaukee, WI): intravenous injection of 3 MBq/kg (0.08 mCi/kg) of ^18^F-FDG, then no contrast enhanced CT acquisition (native collimation 16 × 1.25 mm, auto mA mode with ASIR) and PET acquisition (midthigh-skull base) with 3-dimensional (3D) time of flight mode: 6 to 7 step of 2 to 3 minutes. PET images were reconstructed using an iterative algorithm (OSEM: 24 subsets and 2 iterations) with correction of impulsional response on a 256^2^ matrix with a reconstructed slice thickness of 3.27 mm. A postfiltering Butterworth filter was applied (6.4 mm cut-off frequency).
- Philips Gemini Dual (Philips Healthcare, Eindhoven, the Netherlands): intravenous injection of 5 MBq/kg (0.1 mCi/kg) of ^18^F-FDG, then no contrast enhanced CT acquisition (2 protocols according to morphology: 120 kV to 100 mAs and until 140 kV to 150 mAs, 6.5 mm slice thickness) and PET acquisition (midthigh-skull base) 3 minutes by step. PET images were reconstructed using an iterative algorithm (3D-RAMLA).

### 2.3 ^18^F-FDG PET/CT analysis

The images were interpreted on the basis of a visual analysis, by a nuclear medicine physician (1 reader) aware of the clinical history of the patient on a dedicated display console (AW Server; General Electric). All available ^18^F-FDG PET/CT (including baseline and follow-up scan) were reviewed to assess patient outcome.

All well individualized lung injuries regarding metabolism (greater ^18^F-FDG uptake than in the normal lung parenchyma) and/or on the CT (lung opacities, interstitial lesions, effusion, and nodules) were analyzed. The international nomenclature of bronchopulmonary segmentation was used to localize and quantify pulmonary anomalies (Table 1). To quantify the number of lung segments involved: a partial involvement of lung segment (less than or equal to half a segment) counted for 0.5 and the involvement of a partial segment greater than half a segment or a complete segment counted for 1. Then, a per segment analysis was performed by adding all lung involvements in the same segment for all available ^18^F-FDG PET/CT.

**Table 1:**
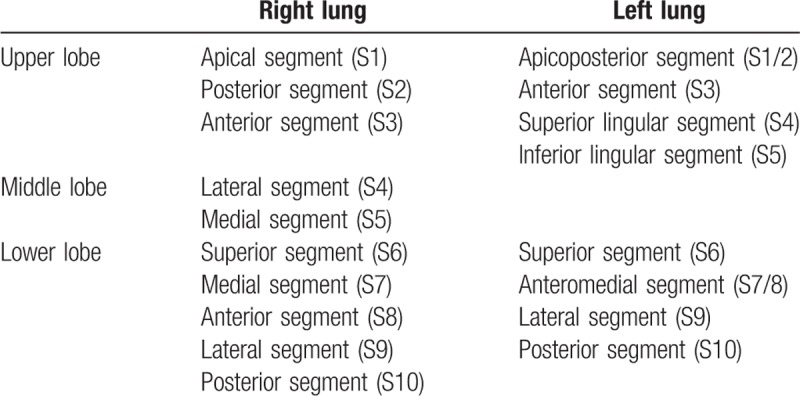
International nomenclature of bronchopulmonary segmentation.

Radiotracer uptake quantification was performed from a volume of interest (VOI) placed manually on each target lesion in attenuation corrected PET images allowing to obtain standardized uptake value (SUV) (classical body weighted SUV). The maximum SUV (SUVmax) was determined as the highest SUV within all voxels included in VOI.

After manual elimination of all extrapulmonary or tumoral lesion uptake, metabolic target volume (MTV) was obtained by summing all voxels with a SUV above a threshold of 42% of SUVmax.^[16]^ Total lesion glycolysis (TLG), defined as the MTV multiplied by the average SUV (SUVmean) of the same voxels, was also obtained.

A retrospective analysis of computerized patient medical files was performed to determine the apparition of pneumopathy (interstitial or infectious) retained by the referring oncologists (most frequently relying on dyspnea, fibroscopy, bacteriologic sample) corresponding to the gold standard in our study.

A Wilcoxon statistical test was performed using R software.

Ethical approval was not considered necessary for this retrospective observational study, as it was based exclusively on data extracted from patients’ medical files. However, principles of the Declaration of Helsinki were followed, and the study was performed in compliance with good clinical practice.

## 3 Results

Twenty-nine patients were included in our study, representing a total of 57 ^18^F-FDG PET/CT performed in average 6 months (2–18 months) after initiation of treatment. Characteristics of patients and lung anomalies are summarized in Table 2.

**Table 2:**
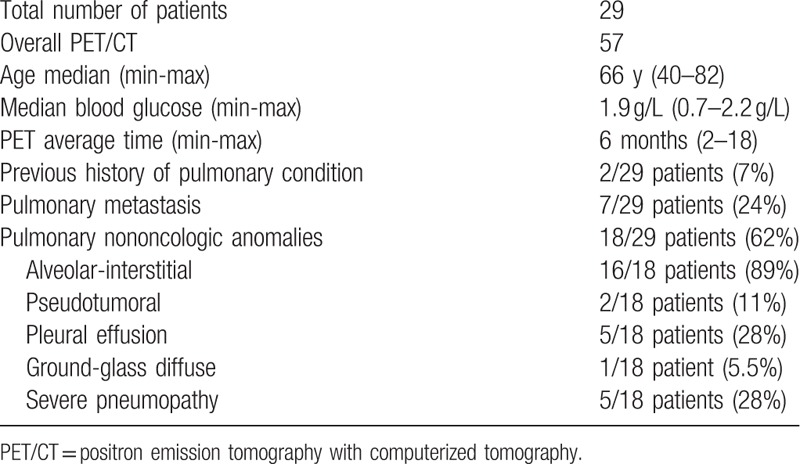
Characteristics of patients and lung anomalies.

### 3.1 Adverse effects

Among 29 patients included, 24 developed toxicities including: 62% lung lesions, 62% digestive disorders (colitis, stomatitis, anorexia, diarrhea, nausea), 29% asthenia, 25% skin diseases (rash, wound, and onychodysplasia), 20% metabolic disorders (hyperglycemia, dyslipidemia), 1 case of cardiac decompensation and 1 case of hepatitis (drug interaction). Most of these toxicities were low grade (grade I or II). Five patients exhibited severe pneumopathy, 3 patients grade III stomatitis, and 1 patient severe colitis.

### 3.2 Characteristics of lung lesions

#### 3.2.1 Radiologic presentation

- 16/18 patients (89%) developed alveolar-interstitial lung disease with multifocal organized and well segmented areas of alveolar lung infiltration and reticular opacities associated with ground-glass opacities, often bilateral, but not necessarily symmetric.
- 2/18 patients (11%) had pseudotumor nodules.
- 5/18 patients (28%) had pleural effusion (all were low abundance effusions).
- 1/18 patient (5.5%) had ground-glass diffuse opacities with no other CT abnormality.

#### 3.2.2 Number, repartition and variation with time of involved segments

In the 16 patients who developed alveolar-interstitial lung disease (corresponding to 26 ^18^F-FDG PET/CT), pulmonary lesions involved 2.8 segments (0.5–11.5) with a median of 2 segments. Left and right S7 and S10 were the most involved segments. Table 3 summarizes the repartition of alveolar-interstitial involvement per segment (sum of all segment involved on ^18^F-FDG PET/CT images) and Table 4 indicates their variation in time.

**Table 3:**
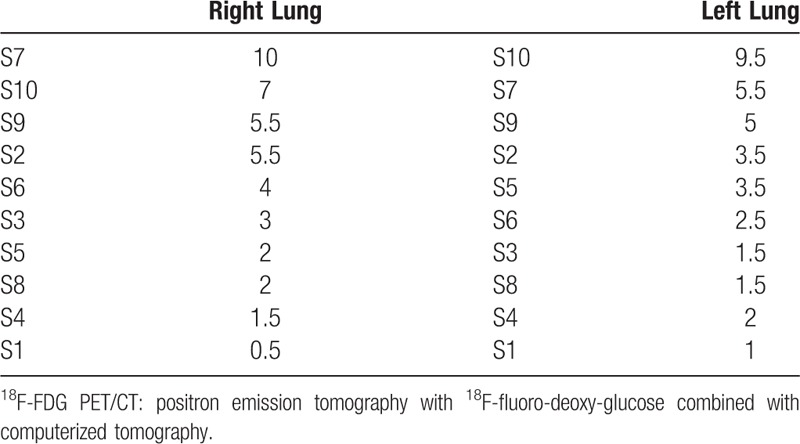
Repartition of alveolar-interstitial involvement per segment in 26 pathologic ^18^F-FDG PET/CT.

**Table 4:**
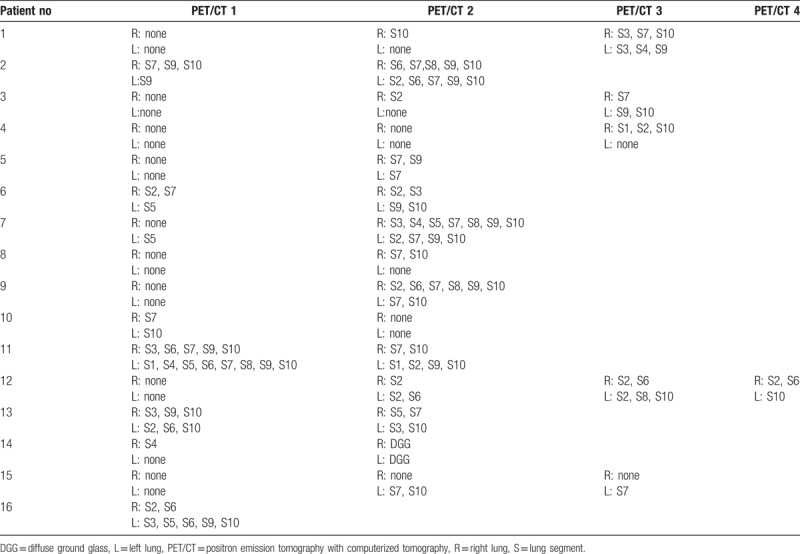
Evolution of alveolar-interstitial lung disease.

#### 3.2.3 Metabolic presentation of alveolar-interstitial lesion

Considering all patients who developed alveolar-interstitial lung disease (corresponding to 16 patients and 26 ^18^F-FDG PET/CT), SUVmax was 3.9 (1.3–8.8) and SUVmean was 2.2 (0.7–4.9). Among 19 pathologic ^18^F-FDG PET/CT performed with Discovery 710, SUVmax, and SUV mean were 4.1 (1.4–8.8) and 2.4 (0.8–4.9), respectively. Among 7 pathologic ^18^F-FDG PET/CT performed with Philips Gemini, SUVmax, and SUVmean were 3.3 (1.3–4.7) and 1.8 (0.7–2.44), respectively.

Two patients presented alveolar-interstitial opacities without PET anomaly (1 with diffuse ground glass).

#### 3.2.4 Subgroup analysis: severe pneumopathy vs interstitial lung disease

Among 29 patients included, 5 patients developed severe pneumopathy (inflammatory or infectious), representing 17% of our overall population. SUVmax, SUVmean, MTV, TLG, and number of segment involved were analyzed to characterize severe pneumopathy. Results of this subgroup analysis are reported in Table 5.

**Table 5:**
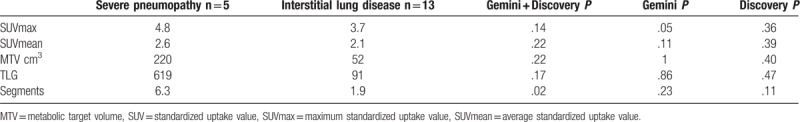
Subgroup analysis between severe pneumopathy and interstitial lung disease.

#### 3.2.5 SUVmax and SUVmean

SUVmax and SUVmean were 4.8 (3.7–7.8) and 2.6 (1. 9–4.3), respectively, in severe pneumopathy group vs 3.7 (1.3–8.8) and 2.1 (0.7–4.9), respectively, in interstitial lung disease group. No statistically significant difference was found (*P* = .14 and *P* = .22, respectively).

#### 3.2.6 MTV and TLG

The MTV mean and TLG mean were 220 (20–717) and 619 (48–1749), respectively, in severe pneumopathy group vs 52 (6–245) and 91 (9–507), respectively, in interstitial lung disease group. No statistically significant difference was found (*P* = .22 and *P* = .17, respectively).

#### 3.2.7 Number of segment

Severe pneumopathy involved an average of 6.3 segments (2.5–11.5) vs 1.9 segments (0.5–8) for interstitial lung disease. This difference was statistically significant (*P* = .02).

## 4 Discussion

### 4.1 Pulmonary adverse effects rate and severe pneumopathy rate

In our study, the rate of lung anomalies detected by ^18^F-FDG PET/CT was 62% in patients treated with everolimus. It is slightly higher than those found in the literature (39–54%),^[11,17,18]^ because we decided to include all pleuro-parenchymal anomalies. Indeed, definition of pulmonary adverse effect was not well defined and varies between studies (interstitial lung disease, pneumonitis, etc). Also, only 29 patients met inclusion criteria (metastatic breast cancer initially treated by everolimus–exemestane who underwent at least one 18F-FDG PET/CT) potentially leading to a selection bias, which could lead to higher observed frequency of pulmonary toxicities among our cohort. However, if we focus only on alveolar-interstitial opacities, the rate falls to 55% and close to those of others studies.

### 4.2 Radio-metabolic presentation of lung anomalies

**Figure 1:**
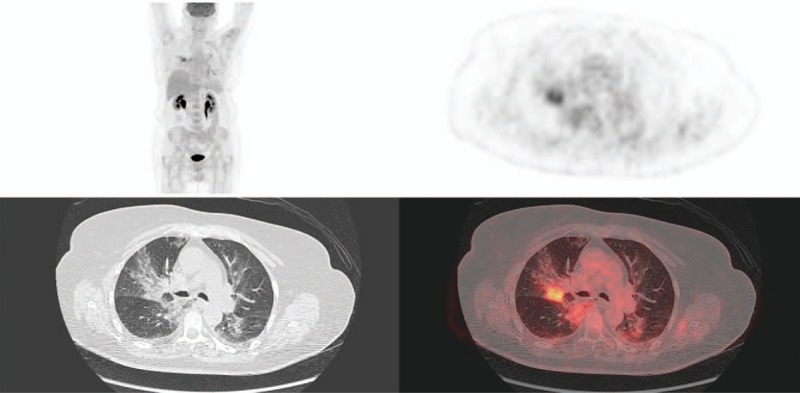
Positron emission tomography with 18F-fluoro-deoxy-glucose combined with computerized tomography (^18^F-FDG PET/CT) (MIP, axial slices, PET, CT, and fused). Classical alveolo-intertitial lung opacities in posterior segment of right upper lobe (S2) and superior segment of right lower lobe (S6) with moderate uptake.

In our study, pulmonary side effects presented most of the time as alveolar-interstitial opacities (89%), sometimes with focal ground glass opacities areas and septal thickening pattern, sometimes with nonspecific diffuse interstitial pattern or organized pneumonia-like pattern (Fig. 1). Alveolo-interstitial opacities were most of the time related to moderate uptake on ^18^F-FDG PET/CT, with SUVmax ranging from 1.3 to 8.8. The differential diagnoses of inflammation, infection or neoplasia in this population of metastatic patients are not always easy. Like conventional imaging, ^18^F-FDG radiotracer lacks of specificity because interstitial lung disease may be sometimes superinfected by unusual germs (*Pneumocystis carinii*, *Aspergillosis*, *Candida*), making it impossible to distinguish between infection and inflammation.^[11]^ When persistent doubt, it could be necessary to conduct additional examinations, especially bronchoalveolar lavage. Furthermore, an analysis by segment shows that the lower lobes are most often affected, especially the median (S7) and posterior segments of lower lobes (S10) and to a lesser importance the lateral segment of lower lobe and posterior segment of superior lobe (Table 3). In our study, ^18^F-FDG PET/CT detected pulmonary toxicity affected more often the lower lobes, like with conventional imaging.^[12]^ Extent and location of involved segments varied with time (Table 4), some appeared with time and others disappeared. That is why we choose to sum all segments.

**Figure 2:**
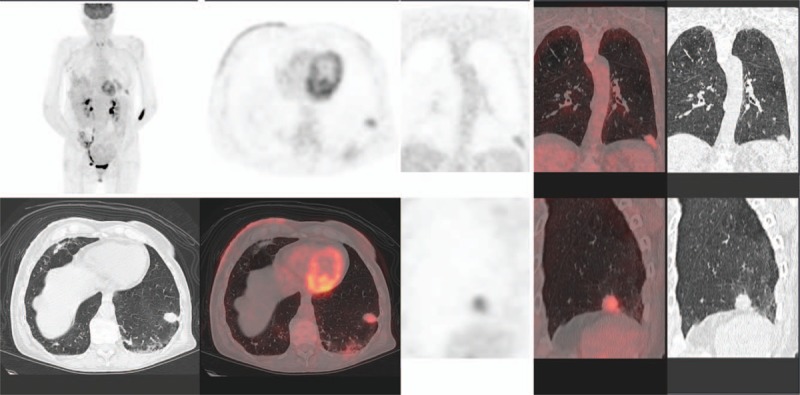
Positron emission tomography with 18^F^-fluoro-deoxy-glucose combined with computerized tomography (^18^F-FDG PET/CT) (MIP, axial, coronal, and sagittal slices, PET, CT, and fused). Pseudonodular presentation of a lung lesion of the lateral segment of the left lower lobe (S9) with moderate uptake (SUVmax = 4.7): a pitfall.

More rarely, interstitial disease could take a pseudotumoral aspect (Fig. 2) which can result in wrong disease progression diagnosis and in unnecessary change in treatment strategy. However, in this presentation, metabolic activity of inflammatory lesions remained moderate (SUVmax < 5 for the 2 lesions in our study). Metastasis presented usually as well delimited nodules with high uptake. Tumoral marker blood test are usually useful in this situation, but CA15.3 marker can be influenced by lung inflammation.^[19]^ Noninfectious pneumonitis can also be difficult to distinguish from lymphangitic tumor spread. In these cases, bronchoscopic biopsies or repeated imaging studies after interruption of everolimus might be helpful.^[20]^

Note that 2 patients presented alveolar-interstitial opacities without uptake (1 with diffuse ground glass and another with alveolar-interstitial opacities of small size), corresponding to false negatives due to a very small size of lesion.

Furthermore, other lung anomalies induced by everolimus were also described in different study, but no visualized in our study, for example, traction bronchiectasis, honeycombing, air trapping, and pericardial effusion.^[11]^

### 4.3 Quantification

#### 4.3.1 SUVmax and SUVmean

Alveolar-interstitial lesions had most of the time moderate uptake: average SUVmax = 3.9 (1.3–8.8) and SUVmean = 2.2 on average (0.7–4.9). These measures appeared similar between both camera used in our study. SUVmax or SUVmean were very close and the superior and inferior limits overlapped for lung anomalies detected with Discovery 710 and Philips Gemini Dual.

SUVmax and SUVmean were not able to distinguish severe pneumopathy and interstitial lung disease (*P* = .14 and *P* = .22, respectively). Taking into account 2 different PET cameras, no statistically significant difference was found, which may be due to the small sample size.

#### 4.3.2 MTV and TLG

**Figure 3:**
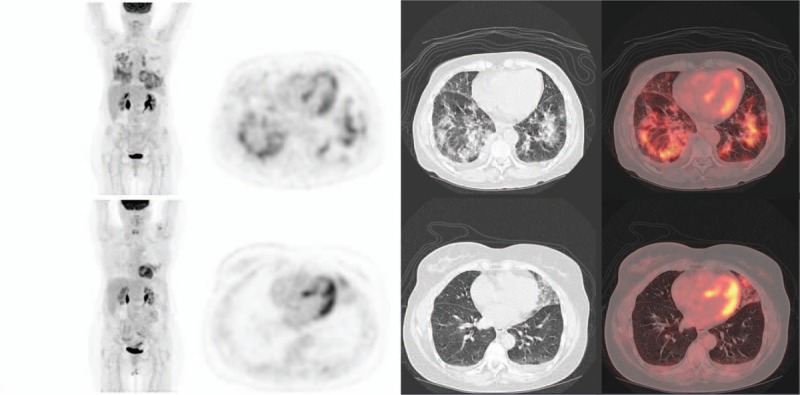
Positron emission tomography with 18^F^-fluoro-deoxy-glucose combined with computerized tomography (^18^F-FDG PET/CT) (MIP, axial slices, PET, CT, and fused). Bottom line: limited area of alveolo-interstitial lung disease with moderate uptake in the inferior lingular segment (S5) 3 months after initiation of everolimus–exemestane association. Top line: apparition of diffuse lung anomalies corresponding to a severe pneumopathy 6 months after therapy initiation.

In our study, 5/29 patients (17%) developed severe pneumopathy (inflammatory or infectious) during treatment, more than in the results of a meta-analysis with patients treated by mTOR inhibitors, in which the incidence of high grades pneumonitis (grades 3 and 4) was 2.4%.^[21]^ The radiologic presentation in computed tomography was typically diffuse nonspecific alveolar-interstitial pneumonitis (Fig. 3). Quantification analysis showed a tendency to differentiate patients with severe pneumopathy vs interstitial lung disease by measures of MTV and TLG, but no statistically significant differences was observed (*P* = .22 and *P* = .17, respectively), probably due to the small patient sample sizes in our study. The number of involved lung segments could be useful (*P* = .02). We keep in mind that evaluation of involved pulmonary segments is different from MTV obtained in cm^3^ by semi-automated segmentation method (42% threshold), possibly leading to discordant results between these measures. Moreover, ^18^F-FDG PET/CT was not always performed during episode of severe pneumopathy, which could underestimate the usefulness of quantitative index.

## 5 Conclusion

The efficacy validation of mTOR inhibitor in oncology (renal cancer, pancreatic NETs, breast cancer, etc) naturally leads to increase their use,^[22–25]^ especially everolimus, an oral therapy usually well tolerated in comparison with cytotoxic chemotherapy.^[26]^ Physicians need to recognize everolimus toxicities, given is efficacy in metastatic breast cancer. ^18^F-FDG PET/CT could evaluate the therapeutic efficacy in patients with metastatic breast cancer treated with everolimus–exemestane and highlight pulmonary side effects, with consequences in the personalized care of these patients. Detection of hypermetabolic interstitial lung disease on ^18^F-FDG PET/CT in breast cancer patient with rapamycin derivative treatment corresponds to a common side effect (62% in our study). A typical imaging pattern (alveolar-interstitial opacities associated with moderate uptake, more or less extensive, mainly affecting the lower lobes) contrasting with poor clinical symptoms is more characteristic of inflammatory lesions caused by everolimus, but rarely a pseudotumoral aspect may be detected, leading to wrongly diagnose disease progression, corresponding to a pitfall which must kept in mind. Sometimes, an important pulmonary involvement may be observed and must to rule out severe pneumopathy and manage treatment. These pulmonary radiometabolic changes are not specific and it is not possible to differentiate between inflammation or infection based on imaging alone, justifying a specialized pneumology consult, bronchoscopy, and pulmonary function evaluation. A poor clinical tolerance (dyspnea, hypoxia, cough, fever, hemoptysis, etc) could require to change dosage of everolimus (decreased to half dose, therapeutic pause, or even premature end) or introduction of corticoid according to observed clinical tolerance.^[27]^ Symptoms are often reversible when this therapy is stopped, but careful monitoring is required with everolimus, because grades 3 to 4 pulmonary toxicity can be life threatening.^[28]^

A good knowledge of the different radio-metabolic presentations of these pulmonary effects would allow better management and avoid potential diagnostic pitfalls. Although our study included limited number of patients to draw strong conclusions, MTV or TLG showed a tendency to differentiate patients with severe pneumopathy vs interstitial lung disease but no statistically significant differences was observed contrarily to the number of segments involved. Further studies are necessary to investigate usefulness of quantification indexes and to determine if the ^18^F-FDG PET/CT could early predict adverse effects of everolimus and mTOR inhibitors.

## Author contributions

**Data curation:** Jean-Christophe Eymard, Gabriel Yazbek, Aude-Marie Savoye.

**Methodology:** David Morland.

**Supervision:** David Morland, Dimitri Papathanassiou.

**Visualization:** Claire Bruna-Muraille.

**Writing – original draft:** Sebastien Dejust, David Morland.

David MORLAND orcid: 0000-0001-8738-4841
